# Hallmarks of senescence and aging

**DOI:** 10.11613/BM.2019.030501

**Published:** 2019-08-05

**Authors:** Slavica Dodig, Ivana Čepelak, Ivan Pavić

**Affiliations:** 1 Department of Medical Biochemistry and Hematology, Faculty of Pharmacy and Biochemistry, University of Zagreb, Zagreb, Croatia; 2 Department of Pulmonology, Allergology and Immunology, Children’s Hospital Zagreb; School of Medicine, University of Zagreb, Zagreb, Croatia

**Keywords:** senescence, aging, biomarkers, hallmarks

## Abstract

The complex process of biological aging, as an intrinsic feature of living beings, is the result of genetic and, to a greater extent, environmental factors and time. For many of the changes taking place in the body during aging, three factors are important: inflammation, immune aging and senescence (cellular aging, biological aging). Senescence is an irreversible form of long-term cell-cycle arrest, caused by excessive intracellular or extracellular stress or damage. The purpose of this cell-cycles arrest is to limit the proliferation of damaged cells, to eliminate accumulated harmful factors and to disable potential malignant cell transformation. As the biological age does not have to be in accordance with the chronological age, it is important to find specific hallmarks and biomarkers that could objectively determine the rate of age of a person. These biomarkers might be a valuable measure of physiological, *i.e.* biological age. Biomarkers should meet several criteria. For example, they have to predict the rate of aging, monitor a basic process that underlies the aging process, be able to be tested repeatedly without harming the person. In addition, biomarkers have to be indicators of biological processes, pathogenic processes or pharmacological responses to therapeutic intervention. It is considered that the telomere length is the weak biomarker (with poor predictive accuracy), and there is currently no reliable biomarker that meets all the necessary criteria.

## Introduction

In the past two decades the field of both aging and senescence research has undergone a significant progress. Aging can be defined as the time-relating irreversible proliferative deterioration of those physiological processes of the organism that support its survival and fertility (*1*). The result of aging processes is the progressive loss of physiological integrity and impaired function of tissues and organs. With prolonged human lifespan, aging also moves towards the older age. Recently, elderly age was classified into three periods: elderly or early old age, senile or middle old age and late old age (or long-livers). Early old age ranging from 60 to 75 years is the period of initial involution of human physical capabilities. Then follows the middle old age, from 76 to 90 years, the period of further involution of human motor functions. Finally, after 90 years of age, a late old age is following; it is a period of decline in human physical abilities (*2*).

Every living organism lives in a permanent struggle with extrinsic and intrinsic agents that can damage it. Without its own repair mechanisms, life of living creatures would be extremely short, since the accumulation of harmful substances would damage the cellular elements, their function, which would ultimately result in damage to the various tissues and accelerated aging of the entire organism.

Most of the aging definition involves a gradual, heterogeneous impair in the structure, function, and maintenance of repair systems of various organs and an increased inclination to various diseases. One could say that the age/aging phases are easy to recognize, but the mechanisms responsible for the aging process are difficult to define and harder to prove. Technological progress has established various methodological approaches to detect some cellular and molecular mechanisms associated with aging. Among others, scientists have focused recently on senescence (cellular aging, biological aging) mechanisms as one of the key factor in a complex aging process (*3*, *4*).

This review focuses on human senescence and aging processes, and their mechanisms. Particular attention was directed to hallmarks of these processes and their possible biomarkers. In search of scientific and review papers on the PubMed free search engine, the following key words were used: lifespan, aging, systems biology, senescence, hallmark, markers of aging, biomarkers, biomarkers of senescence, senescence testing, and bioinformatics. Epidemiological and clinical researches were studied primarily on older people, regardless of their ethical affiliation. Also, animal models of aging investigation were studied. Abstracts, reports from meetings and case control studies were excluded. Articles published in English between 1997 and 2019 were included. Articles were selected according to relevance to the topic.

**Figure 1:**
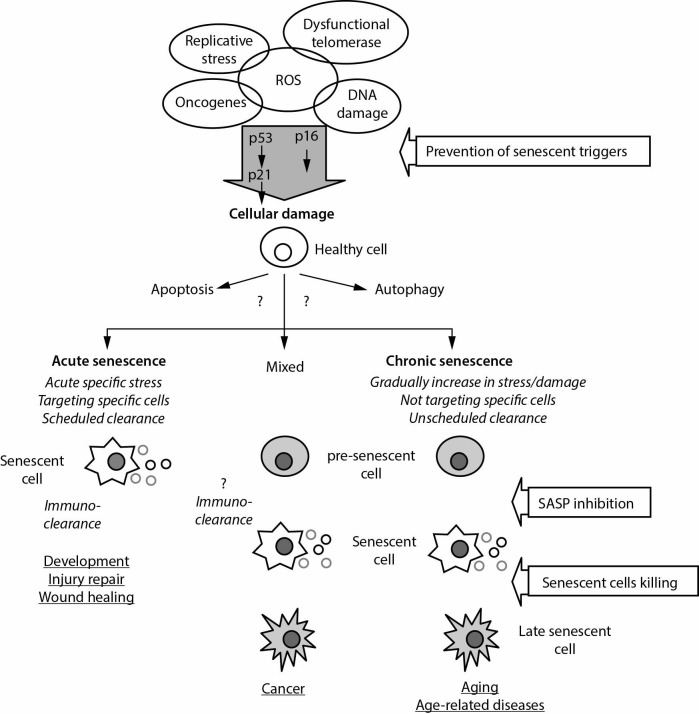
Overview of the process of senescence and its contribution to aging of entire organism (adapted according to references 5, 10 and 11). Based on kinetics of cell senescent processes there are two main categories of senescence – acute (programmed, transient) and chronic (not programmed, persistent) senescence. While acute senescence leads to embryonic development, wound healing and tissue repair of specific populations of cells and tissues, chronic senescence that is not directed towards specific cells leads into a stable cell-cycle arrest, a state that limits the proliferation of damaged cells. The main mediator of acute senescence is SASP. It seems that, because of age-related immunodeficiency or less production of proinflammatory SASP factors, immune cells becomes inefficiently in the elimination of senescent cells. p53, p16 and other tumour suppressor pathways mediators leads to senescence. Cancer development will occur if pre-senescent cells (stressed cells) would not been removed by specific mechanisms. However, it is not known which mechanisms are responsible for direction to senescence, apoptosis or to autophagy. Production of SASP factors may be inhibited by the use of: nuclear factor kappa-light-chain-enhancer of activated B cells (NFκB), interleukine 1α blockers, rapamycin, metformin; senescent cell killing may be induced by natural killer cells, T cell targeting, antibodies or antibody-mediated drug delivery. Early in life, senescent cells are transiently present and have a beneficial effect on development, homeostasis, and regeneration. However, at a later age, senescent cells accumulate and produce detrimental effects. ROS – reactive oxygen species. SASP – senescence-associated secretory phenotype. p53 – cellular tumour antigen p53. p21 – cyclin-dependent kinase inhibitor 1, cell-cycle inhibitor. p16 – cyclin-dependent kinase inhibitor 2A, multiple tumour suppressor 1.

Three different responses that have protective role in response to cellular stressors are apoptosis (programmed cell death), autophagy (from the Greek noun „autóphagos“, meaning self-devouring) and senescence (irreversible arrest, that limits the proliferation of damaged cells) (*5*-*8*). It seems that the cellular response depends on the type of cell that is subjected to the harmful effect of the stressor. While damaged lymphocytes tend to undergo apoptosis, damaged epithelial cells and fibroblasts tend to undergo senescence (*5*). Autophagy implies a lysosome-mediated cell’s own components bulk degradation and clearance (*5*, *9*). The relationship between autophagy and apoptosis is complex. It is not yet clear which factor determines whether cells will die with apoptosis or with other mechanisms. It seems that autophagy could be conducive to cell death in cases when apoptosis is inhibited (*5*). While activation of autophagy causes inhibition of apoptosis, its inhibition increases susceptibility to oxidative damage of the cell and apoptosis. Prolonged autophagy is associated with cell death. Autophagy becomes defective during ageing and especially in patients with age-related diseases, since degraded molecules and organelles accumulate in cells. Hence, defective autophagy is a feature of old cells (*7*). Schematic depiction of the aging process, with possible therapeutic interventions is shown in Figure 1.

## Senescence

Senescence (from the Latin word „senex“, meaning growing old) is an irreversible form of long-term cell-cycle arrest, caused by excessive intracellular or extracellular stress or damage (*12*). In order to avoid malignant transformation after the stressor’s activity, cellular senescence refers to the arrest in the G1 phase of the cell-cycle (*5*). Senescent cells are however functionally and metabolic active as changes occur, for example change of degradation pathways of proteins, enhanced mitochondrial metabolism, energy generation, *etc.* (*13*). The purpose of senescent cells arrest is to limit the proliferation of damaged cells (*e.g.* the spread of damage to the next cell generation), to eliminate accumulated harmful factors and to disable potential malignant transformation (*5*-*8*). In young tissues, transient senescence has beneficial effect. The good example is the beneficial effect of senescence to pregnancy that implies proper foetal development and time of parturition. A detrimental effect refers to reproductive capacity since it causes the decrease in the number of ovarian follicles, and in later age senescence causes decline in ovarian and uterine function (*14*). Healthy senescence may be accelerated by elevating the concentration of oxygen or various toxic substances (*15*). Factors that slow down damage accumulation delay the senescence.

Based on kinetics of cell senescent processes there are two main categories of senescence, *i.e.* acute (transient) and chronic (persistent) senescence (*16*). Acute senescence is the part of normal biological processes, and has beneficial effect within tissues during embryonic development, wound healing or tissue repair. Myofibroblasts have an important role during acute senescence, because they promptly undergo senescence, limiting excessive fibrosis at the site of cell/tissue damage. Acute senescence may be a part of programmed mechanism of fibrosis control during tissue repair (*17*). Acute senescent cells are eliminated through activation of senescence-associated secretory phenotype (SASP) factors and consequently activated immune clearance. Senescent cells, still metabolically active, found primarily in tissues with chronic inflammation and in renewable tissues, are able to create an inflammatory microenvironment, to recruit phagocytic cells for elimination of senescent cells and finally, to promote tissue removal. They secrete a variety of different molecules to communicate with adjacent cells. Senescence is enabled with the acquisition of SASP factors, such as interleukins (the most prominent is interleukin-6, IL-6), chemokines, growth factors (*e.g.* insulin-like growth factor, IGF) and regulators, proteases (*e.g.* matrix metalloproteinases - MMPs, serine proteases), *etc.* (*8*, *18*, *19*). Released SASP factors are involved in sensitizing non-senescent neighbouring cells to senesce, cell proliferation, disruption of normal tissue structure and function, immunomodulation (immune cells clearance), angiogenesis, disabling or fostering of cancer growth. SASP factors have beneficial role during embryogenic development, accelerating wound healing, after tissue injury (by limiting fibrosis), involved in the amplification and spread of senescent cells, during suppression of tumorigenesis by promoting the elimination of senescent cells. The main function of SASP is to eliminate senescent cells. If there were no senescent cell clearance as in case in elderly people, senescent cells would accumulate, which would have detrimental consequences implying structural, degenerative, irreparable tissue damage and fibrosis (*7*, *20*). Chronic senescence is induced through prolonged period of cellular stress or slow macromolecular damage (*10*, *16*). Complex effector pathways involved in chronic senescence significantly differ from pathways in acute senescence, due to large SASP heterogeneity involved in chronic processes and high resistance of senescent cells to immune clearance. Chronic senescence has detrimental effects within cells and tissues. The knowledge that senescence can cause age-related diseases has instigated researchers to develop drugs that can eliminate senescent cells. These medications could improve health in the elderly (Figure 1) (*11*, *20*).

Senescent cells in elderly are not able to maintain neither physiological tissue functions nor tissue repair, including autophagy, whose capacity declines with aging (*7*, *21*, *22*). Cellular senescence is followed by senescent cell clearance within those processes that are considered beneficial. However, if the elimination of senescent cells does not occur, senescent cells accumulate and can lead to cancer and aging. Investigations on animal samples have shown that senescent cells accumulate in old animals in leukocytes and intestinal crypt enterocytes, in dermal fibroblasts, hepatocytes, osteocytes (*23*).

Unlike apoptosis in which phagocytes remove cells without causing inflammation, senescent cell survive because of stimulation of the inflammatory environment and removal of harmful compounds (*24*). Senescence-associated beta-*galactosidase* (SA-β-GAL), is an isoform of the beta-galactosidase enzyme, normally responsible for the breakdown of beta-galactosides. Its activity is present in lysosomes of senescent cells. Increased activity of SA-β-GAL is considered to be an outcome of senescence (*7*).

### Factors leading to senescence

Senescence can be triggered *e.g.* by oxidative stress, telomere damage/shortening, DNA damage, mitochondrial dysfunction, chromatin disruption, inflammation, epigenetic dysregulation, and oncogene activation (*17*, *25*-*27*).

#### Oxidative stress

It is known that senescent phenotype may be stimulated/induced by various types of stresses, including that induced by reactive oxygen species (ROS). Reactive oxygen species are a natural by-product of the normal oxygen metabolism. It is considered that ROS regulate several physiological functions, like signal transduction, gene expression and proliferation. The major cellular sources of ROS are mitochondria, cell membranes and endoplasmic reticulum (*28*). While lengthening of organismal lifespan is associated with low ROS concentration, senescent phenotype maintenance is endangered with high ROS concentrations (*29*). The oxidant/antioxidant imbalance causes a structural damage of macromolecules (DNA, proteins and lipids). Age-related accumulation of damaged macromolecules is one of mechanisms that contribute to the aging processes. The balance between oxidant generation and antioxidant processes in healthy tissues is maintained with a predominance of various antioxidants (*30*, *31*).

Reactive oxygen species of endogenous or exogenous origin induce and firm the senescent phenotype by a process that involves the response to DNA damage, epigenetic regulation and tumour suppression pathway activation (*e.g.* cell cycle control related proteins: p53 (cellular tumour antigen p53), p21 (p21^Cip1^, cyclin-dependent kinase inhibitor 1), pRB (retinoblastoma protein). These mechanisms, more specifically SASP factors of senescent cells, on the other hand, can stimulate positive feedback loop and result in increased ROS, especially mitochondrial ROS (mtROS) (*32*). As mitochondria are the main place of ROS creation, investigations have shown that mitochondrial dysfunction is associated with senescence, and consequently with the aging process. It is considered that mtROS and oxidative stress in general can stimulate telomeres shortening and dysfunction, which is one of the characteristics of aging (*33*). In addition to ROS, as senescence inducers, other mitochondrial-related effectors are also considered, for example, redox changes, changed metabolism (*34*, *35*).

#### Telomere shortening

Telomeres (from the Greek nouns „telos“ meaning end and „merοs“ meaning part), specialized DNA-protein structures of human chromosomes, composed of several kilobases (kb) of simple repeats (TTAGGG)n are located at the ends of chromosomes. The length of telomeres is an accurate predictor of the replicative ability of cells. The basic function of telomeres is to protect the chromosomes from degradation rearrangements, end-to-end fusions, and chromosome loss (*36*). Shortening occurs at each cellular division but is counteracted by telomerase. Telomerase is an enzyme complex that maintains telomere length. It is considered that telomeres participate in the protection of ends of chromosomes from constitutive exposure to the DNA damage response (*37*). Telomere length progressively shortens with replication of nuclear DNA during mitosis, or with oxidative stress or with senescence and aging (*38*). While the length of the telomere at birth is about 11 to 15 kb in elderly it is significantly shorter, about 4 kb (*39*-*42*). So, senescence is mostly triggered when the length of the telomere shorten from 5–20 kb to 4–7 kb (*43*). The shortening of the telomeres that occurs during normal aging is controlled by the activity of specialized enzyme telomerase (*27*). However, the balance between telomere shortening and counteracting by telomerase is disrupted during accelerated senescence as a result of the disease.

#### DNA damage

Critically short telomeres are recognized as DNA damage, which trigger a DNA damage response (DDR). The DDR arrests cell cycle progression until damages are repaired. However, senescent cells display persistent DDR foci that that are resistant to endogenous DNA repair (*44*).

#### Mitochondrial DNA damage

Mitochondria are intracellular source of oxygen. Functional mitochondria regulates cellular homeostasis through the maintenance of redox balance, which implies a balance between oxygen uptake, ATP production, membrane potential and generation of ROS (*45*). Mitochondria that accumulate in senescent cells show increased concentrations of ROS and increased rate of senescent cells in the same tissues, resulting in mitochondrial dysfunction (*27*, *45*).

#### Tumour suppressors and cell cycle inhibitors

Today, several suppressors and cell cycle inhibitors are known, *e.g.* p16 (known as cyclin-dependent kinase inhibitor 2A, multiple tumour suppressor 1), p53, p21, p15 (p15^INK4b,^ protein kinase; cyclin-dependent protein serine/threonine kinase inhibitor, multiple tumour suppressor), p27 (cyclin-dependent kinases regulator), ADP-ribosylation factor (ARF), hypophosphorylated retinoblastoma protein (*7*, *11*). Activation of the tumour suppression pathways p53 and p21 and the p16/retinoblastoma protein pathways occurs during senescence. Activation is triggered by the DNA damage, which may be result of telomeric and non-telomeric DNA damage or oxidative stress (*27*).

### Characteristics of senescent cells

Senescent cells are characterised by flattened and enlarged morphology. They exhibit several molecular markers, including telomere-dysfunction-induced foci, senescence-associated heterochromatin foci (SAHF), lipofuscin granules, DNA scars, altered gene expression (*5*, *7*). Another important feature of senescent cells is release of SASP factors (*19*). As the senescent cells are characterized by the irreversible growth arrest in either G_1_ or G_2_/M phase of the cell cycle, they are no longer able to divide. These cells have special biochemical characteristics, *e.g.* the absence of proliferative Ki-67 protein, activity of senescence-associated β-galactosidase (SA-β-GAL), expression of tumour suppressors and cell cycle inhibitors (*7*, *11*). Nuclear and mitochondrial DNA damage accelerate senescence. As long as the repair mechanisms are effective, the cell damage can be repaired. Otherwise, when some of the repair mechanisms fail, damaged DNA will accumulate, obstructing cellular function and causing its senescence. Inducers of senescence, such as telomere shortening, toxic agents or oncogenes, cause the formation of SAHF, that contain heterochromatin-forming proteins, such as heterochromatin protein 1 (HP1) proteins, di- or tri-methylated lysine 9 of histone H3 (H3K9Me2/3) and histone H2A variant (macroH2A) (*46*, *47*). All these cellular characteristics can be considered as hallmarks (or possible biomarkers) of senescence.

### Aging

Aging has been the focus of researchers for many years. Scientists are trying answer two basic questions on biochemical level: „Why does human being (and all living organisms) age?“ and „How do organisms age?“. Consequently, there are a large number of aging theories that are classified in a variety of ways. For example, one of classifications theories includes the evolutionary and causality theories (*48*). Evolutionary aging theories, that are focused on the failure of natural selection to affect late-life traits, refer to programmed aging (assisted death), non-programmed aging and senemorphic aging (maladaptive aging, secondary aging). Causality theories imply the influence of the environmental conditions on cellular senescence and ultimate death. The main role was given to telomeres shortening, free radicals damages, spontaneous errors, glycation end-products (*48*). There are also theories that attempt to explain the aging process itself - on the one hand there are theories considering the senescence as programmed processes; other theories, *e.g.* „DNA damage theory of aging“ are focused on the accumulation of damage as the main cause of biological aging (*22*, *49*).

Aging is an intrinsic feature of all living beings. The complex process of biological aging is the result of genetic and, to a greater extent, environmental factors and time. It occurs heterogeneously across multiple cells and tissues. As the rate of aging is not the same in all humans, the biological age does not have to be in accordance with the chronological age. Many age-associated changes and hallmarks are evident in the human body. The changes associated with old age can be divided into a few categories: normal aging, somatic diseases and multiple chronic conditions, psychological, cognitive and social changes (*50*). Normal aging implies sensory changes (visual acuity, hearing loss, dizziness), muscles weakening and reduced mobility ability, fat changes. At the same time the body increasingly succumbs to some diseases, including hypertension, cardiovascular diseases, diabetes, osteoarthritis, osteoporosis, cancer, and several neurological disorders. In elderly there are several functional changes of respiratory system such as reduction of vital capacity, increased residual volume, reduction of pulmonary diffusion, increased arterial-alveolar oxygen gradient, hypoxia, hypercapnia, increased percent of neutrophil granulocytes, increased ratio of CD4^+^/CD8^+^ cells in bronchoalveolar lavage fluid and decreased level of antioxidant compounds (*i.e.* superoxide dismutase, glutathione, catalase, metal binding proteins, vitamins C and E) (*51*, *52*). In addition, there is a decreased number of functional glomeruli, decreased rate of glomerular filtration and renal blood flow (*53*). Occurrence of electrolytic disturbances (*e.g.* hyper- or hyponatremia) may worse other comorbidities (*54*). Also, there is a decrease in basal metabolism, the change in gastrointestinal system, as well as in the hypothalamic-pituitary-adrenal systems. The later results with low response to stimulation of this axis (*54*). In the background of all the changes that occur during aging are three key factors – inflammation, immune aging and senescence.

### Inflammation and aging

Unlike acute (transient) inflammation in which the causative agents are removed and the damaged tissue is cured, chronic inflammation persists for a long time. During chronic inflammation affected tissues are infiltrated with macrophages and lymphocytes. In addition, fibrous and necrosis of the affected tissue may occur (*18*, *55*). Chronic inflammation is associated with many age-related physiologic or pathophysiologic processes and diseases. In normal, healthy aging, serum concentrations of pro-inflammatory cytokines (IL-1, IL-2, IL-6, IL-8, IL-12, IL-15, IL-17, IL-18, IL-22, IL-23, tumour necrosis factor alpha – TNF-α, and interferon-gamma – IFN-γ) are significantly increased in comparison with younger individuals (*56*-*58*). At the same time, in elderly people concentration of anti-inflammatory cytokines (interleukin-1 receptor antagonist – IL-1Ra, IL-4, IL-10, IL-37, transforming growth factor beta 1 – TGF-β1) are higher than in young persons. The role of anti-inflammatory cytokines is to neutralize pro-inflammatory cytokine activity, reduce chronic inflammation, and thus act protectively on tissues. In the case of healthy aging, a balance between the action of pro-inflammatory and anti-inflammatory mediators has been established. Their imbalance leads to aging of the body and to the development of various age-related pathological conditions (*59*).

### Immune system and aging

The weakening of unspecific innate and highly specific acquired immunity takes place through the aging of human cells (Table 1). The phagocytic function is reduced, while, chemotaxis may be conserved, especially in the presence stimulants of the complement fragment C5a (*57*). The number of macrophage precursors is decreased, the phagocytic function is reduced, neutrophil dysfunction is observed, and naive lymphocytes produce less IL-2, the number of CD8^+^ lymphocyte increases. The senile age is characterized by a high expression of CD25 and FOXP3 (a transcriptional factor that is crucial for the function of Treg cells), and increased number of CD4^+^/FOXP3 lymphocytes, changed T17/Treg ratio. All these changes are responsible for the appearance of inflammatory and autoimmune diseases (*60*). Impaired NK function of natural killers (NK) is associated with an occurrence of infective, atherosclerotic and neurodegenerative diseases. As the thymus exhibits degenerative changes, impaired function of both, B cells and T cells leads to imbalance between inflammatory and anti-inflammatory mechanisms. Frequent infectious diseases in old age are a result of impaired function of the innate and acquired immune system. Immune system fails to clear infectious antigens, infected cells, senescent cells, and malignant transformed cells (*56*, *61*). Immunological changes in elderly, based on the decline of the functional capacity of the immune system, result in reduced resistance to infections, increased appearance of neoplasia, and increased production of auto-antibodies responsible for the occurrence of autoimmune diseases (*62*).

**Table 1:** Features of immune aging

**Cell**	**Features**
**Innate immunity**	
Neutrophils	Reduced phagocytosis and ROS production
Monocytes/Macrophages	Reduced phagocytosis, cytokine and chemokine secretion, reduced generation of NO and superoxide, reduced IFN-γ, inhibited response to growth factors
Dendritic Cells	Reduced phagocytosis and pinocytosis, increased IL-6 and TNF-α production, diminished TLR expression and function
Eosinophils	Reduced degranulation and superoxide production
Cytotoxic lymphocytes	
NK	Reduced numbers, increased reduced numbers, reduced cytotoxicity
NKT	Reduced proliferation
**Acquired immunity**	
B cells	Decreased number, reduced proliferative capacity, increased oligoclonal expansion, reduced surface MHC class II molecule expression, reduced antibody avidity, increased concentration of IgG, IgA and concentration of autoantibodies
T cells	Reduced CD28 expression, accumulation of CD8^+^CD28^-^ T cells, reduced TCR diversity, reduced signal transduction, reduced response and proliferation, increased differentiation of CD4^+^ into Th17 cells
Treg	Increased CD8^+^FOXP3^+^, decreased CD8^+^CD45RA^+^CCR7^+^
ROS - reactive oxygen species. NO - nitric oxide. NK – natural killer cells. NKT – natural killer T cell. Treg – T-regulatory cells. TCR – T-cell receptor. IL – interleukine. IFN – interferon γ. TLR – toll-like receptor. TNF**-**α – tumour necrosis factor α. MHC – major histocompatibility complex. CD – cluster of differentiation. FOXP - transcription (factor) protein. CCR – chemokine receptor. Adapted according to references 63-66.	

As individuals of the same age do not have the same rate of age, there is a need to find specific hallmarks that could objectively determine the rate of age of a person. These biomarkers might be a valuable measure of physiological/biological age. Still, there is no universally accepted definition of a biomarker of aging. Phenotypic hallmarks are non-invasive biomarkers, and easy to obtain (Table 2). Biochemical biomarkers can reflect some of the biochemical mechanisms underlying age status. It would be ideal if quantitative aging biomarkers could specifically determine the biological age (healthy aging) of a person, regardless of the predisposition to disease (accelerated aging) (*67*). In laboratory medicine, organ-specific biomarkers imply determining those biochemical and haematological analytes that point to the diseases of particular organic systems.

**Table 2:** Phenotypic and biochemical hallmarks of aging

**Hallmark category**	**Hallmark subcategory**	**Hallmark**	**Trend during aging**
**Phenotypic**	Anthropometry and physical function	BMI, waist circumference	I
	Facial features	Eye corner slope	D
		Nose width, Mouth width, Noise-mouth distance	I
		Mouth width	I
		Noise-mouth distance	I
**Biochemical**	Nutrient sensing	(S/P) Growth hormone and IGF-1	D
	Protein metabolism	(S/P) Protein carbamylation, *e.g.* homocitruline rate	I
		(Erc) Glycosated hemoglobin	I
		(S/P) Advanced glycation end products N-glycans	I
	Lipid metabolism	(S/P) Lipid profile, free fatty acids, isoprostanes	I
	Oxidative stress	(Erc) superoxide dismutase	D
		(Erc) glutathione, glutathione reductase, glutathione peroxidase	HD
	Hormone, energy	(S/P) Triiodothyronine, cortisol	D
	Inflammation	(S/P) C-reactive protein, interleukin 6	I
**Organ-specific**	Cardiovascular system	(S/P) troponin, natriuretic peptides, endothelin	I
	Lung	(S/P) surfactant protein D	I
		(arterial blood) partial pressure of oxygen	D
	Kidney	(S/U) Glomerular filtration rate	D
		(S/P) creatinine, urea	I
	Liver	(S/P) ALT, AST, GGT, albumin	D
	Reproductive function	(S/P) LH, FSH, DHEA	D
	Oxygen transport	(B) Htc, Hb, MCV, Rtc	D
		(S) erythropoietin, ferritin, hepcidin	D
	Blood clotting	(S/P) D-dimers	I
		(B) platelet count	D
		(Plt) platelet functions	I
		(P) Fibrinogen	I
BMI – body mass index. IGF-1 – insulin-like growth factor 1, somatomedin C. S/P – serum/plasma. Erc – erythrocytes. S/U – serum/urine. B – blood. S – serum. P – plasma. ALT – alanine aminotransferase. AST – aspartate aminotransferase. GGT – gamma-glutamyl transferase. LH – luteinizing hormone. FSH – follicle-stimulating hormone. DHEA – dehydroepiandrosterone. Htc – haematocrit. Hb – haemoglobin. MCV – mean cell volume. Rtc – reticulocytes. I – increased. D – decreased. HD – increased in elderly hypertensive patients treated for their conditions. Adapted according to reference 70.			

### Senescence and aging testing

In order to examine why and how people become old with different rate, it is necessary to define the primary indicators/biomarkers of the healthy aging process. Only in this way it will be possible to distinguish the phenomenon of aging due to the processes caused by various diseases that are commonly associated with the aging process. In this sense, the scientific community is continually investing great efforts in discovering such biomarkers.

In general, a biomarker is defined as any substance, structure or process that can be objectively measured in the body or its products and evaluated as an indicator of normal biological processes, pathogenic processes or pharmacological responses to therapeutic intervention (*68*, *69*). Thus, there are diagnostic, prognostic, predictive and pharmacodynamic biomarkers.

According to the American Federation for Aging Research (AFAR) recommendations, aging biomarkers should meet several criteria. They have to: 1. predict the rate of aging (correlate with aging); 2. monitor a basic process that underlies the aging process (determine “healthy aging”, not the effects of disease); 3. be able to be tested repeatedly without harming the person; 4. be applicable to humans and animals (*70*). However, currently, there is no biomarker that would meet all of these criteria. Scientific papers refer at biomarkers of senescence (or senescent cells) as well as at aging biomarkers. Currently, due to the stated fact that many of the hallmarks do not meet biomarker definition criteria, it may be better to use terms a) hallmarks of senescent cells or hallmarks of aging or b) possible biomarkers of senescence.

Research on why and how the senescence goes on should shed more light on this intriguing process. The corresponding biomarker can be identified either in pro-senescent mechanisms either in anti-senescent pathways. Different methods for detection of senescence in tissue sections or in cultured cells (fibroblasts) are used (Table 3). It is possible to conduct morphological analysis of senescent cells, detection of intracellular SAHFs, determination of cell viability, p21 detection and measuring SA‐β‐GAL activity, the ability of autophagy, cell proliferation, leukocyte absolute telomere length by southern blot analyses of the terminal restriction fragments, by quantitative polymerase chain reaction or quantitative fluorescence in situ hybridization (*71*-*76*).

**Table 3:** Laboratory methods used for determination of possible senescent-cell biomarkers

**Analyte**	**Method**	**References**
morphological analysis	inverted phase-contrast microscope	73
cell viability	tetrazolium reduction, microplate spectrophotometer	71
SASP	ELISA	12,68
SAHF	immunohistochemistry	12
γH2AX	histochemistry	12,68
p16, p53, and p21	histochemistry, immunohistochemistry	12
SA‐β‐GAL	histochemistry, immunohistochemistry, flow cytometry	12,68,79
autophagy	immunoblotting	72
cell proliferation	flow cytometry	73
leukocyte absolute telomere length	southern blot, PCR, FISH	68,75,76
ELISA – enzyme linked immunosorbent assay. SASP – senescence-associated secretory phenotype. SAHF – senescence-associated heterochromatin foci. γH2AX – a type of histone protein from the H2A family, a marker for activation of DNA damage response. PCR – polymerase chain reaction. p16 – cyclin-dependent kinase inhibitor 2A, multiple tumor suppressor 1. p53 – tumour suppressor gene, induces senescence growth arrest *via* activated p21–p53 pathway. p21– cell-cycle inhibitor, induces senescence growth arrest *via* activated p21–p53 pathway. SA‐β‐GAL – senescence-associated β-galactosidase. FISH – fluorescent in situ hybridization.		

A lysosomal hydrolase, SA-β-GAL, normally active at pH 4, in senescent cells is active at pH 6. However, the SA-β-GAL, is present not only in senescent cells but also in presenescent, quiescent or immortal cells (*77*). It may be detected in tissue sections histochemically and immunohistochemically (*12*, *78*). Conventional SA‐β‐GAL staining fails to distinguish between different cell types that can be a source of senescent cells within complex tissues, limiting our understanding of the underlying biological phenomena. (*73*, *79*). As the single parameter is not enough to define with confidence that cells are senescent, SA-β-GAL staining may be combined with additional possible biomarkers, *e.g.* SASP factors, SAHF formation, γH2AX (a type of histone protein from the H2A family, a marker for activation of DNA damage response), p16, p53 (induces senescence growth arrest via activated p21–p53 pathway), and p21 concentrations (induces senescence growth arrest via activated p21–p53 pathway) (*12*). Telomere attrition is the intrinsic property of healthy cellular aging, and is also associated with many age-related diseases, like atherosclerosis, myocardial infarction, heart failure, Alzheimer’s dementia (*76*). For more than a decade telomere length has (most often average leukocyte telomere length) been postulated as a biomarker of human aging (*80*).

These possible biomarkers are detected separately in consecutive sections; it means that multiple possible biomarkers are not determined within the same cells. Although it was confirmed in mouse tissues that most possible markers increase with age, there is still insufficient data that would refer to healthy human tissues (*77*). Telomere length measurement is emerging as a tool that may have implications for prevention, disease monitoring, and intervention development. It has been a subject of debate whether telomere length is a biomarker of aging in specific tissues or for a whole organism, since the aging of different tissues and organs of the human body is not the same (*3*, *81*). Therefore, In human aging, telomere length is a weak biomarker with poor predictive accuracy. Glycans might be a better possible biomarker of chronological and biological age than telomere lengths (*81*, *82*). Histochemical staining of lipofuscin (*i.e.* lipid - containing lysosomal granules) of paraffin sections has been shown to be one of the possible markers of senescence in age-related diseases (*83*, *84*). Recently a new method for the determination of lipofuscin in liquid samples of stressed or damaged cells was introduced (*85*). Mass cytometry method, as a method that combines flow cytometry and mass spectrometry, enables the simultaneous quantification of numerous cellular parameters (SA-β-GAL) at single-cell resolution (*86*). Also, among potential predictors of biological age could be included the degree of methylation of DNA, transcriptomic predictors, proteomic predictors, metabolomics-based predictors, and composite biomarker predictors (*87*).

Additional research is needed to confirm that glycans or some other compounds will meet necessary criteria to be the biomarkers of senescence. In the future, biomarker and therapeutic target candidates will be examined for a follow-up study, which will facilitate longitudinal monitoring of therapeutic interventions on senescence and aging.

Today, the bioinformatics, as an interdisciplinary field of science, helps to analyse and interpret biological data on aging and senescence, including studies of gene expression and comparative and pathway analyses (*88*-*90*). Computational biology of aging refers to a wide range of data, from demographic to genomic transcriptomic, proteomic and metabolomic studies (*88*). CSGene database has been developed for exploring cell senescence genes and to highlight the roles of cell senescence genes in the control of rRNA gene transcription (*89*).

Between 1997 and 2019, PubMed published about 363,000 articles on senescence and aging, and in the first four months of 2019, more than 10,000 articles. In this review, 90 articles have been selected to help us better understand the need to discover the hallmarks and biomarkers of senescence and aging. The knowledge of the mechanisms of senescence and the influence of senescence on aging of organism have evolved due to the development of numerous standard and sophisticated and laboratory methods. Senescence and aging can be observed from different aspects so that this topic can be observed in the context of research of mainly human fibroblasts, leukocytes, cell cultures and animal leukocytes and intestinal crypt enterocytes, dermal fibroblasts, hepatocytes, osteocytes, computational biology methods, the examination of factors involved in the normal pathways of acute and chronic senescence, diseases that can affect the process of senescence, processes that can repair senescence effects (*5*, *7*, *10*, *11*, *16*, *17*, *21*-*23*, *27*, *43*, *50*, *78*, *81*, *88*, *89*), *etc*. In order to successfully investigate these processes, it is necessary to find standardized biomarkers of senescence or the healthy aging of the organism (*70*). It is important to know the extent of determining a particular biomarker to prevent age-related assessment of the entire organism. Standardized biomarkers could also help in the monitoring of therapeutic interventions in the process of senescence, which is one of the goals of examining all aspects of senescence (*11*, *21*).

## Instead of a conclusion

The largest number of study of senescence and aging processes were made on cell cultures and animal models.
The senescence seems to be a critical factor in both the normal aging process and pathologies associated with aging.
There are currently no standardized biomarkers („gold standard“) of cellular aging process or the healthy aging of the organism. Biomarkers described in literature do not meet all criteria of an ideal aging biomarker and actually represent various hallmarks of the aging process.
Most biomarkers currently being examined as senescence or aging biomarkers are related to age-related illnesses rather than the process of healthy aging.
As the effector mechanisms of senescence are neither necessarily specific to senescence nor present in all forms of senescence (the rate of senescence is not the same for all types of cells), the interpretation of existing biomarkers of senescence (for now the hallmarks or possible biomarkers) should be context dependent. Additionally, a combination of multiple biomarkers should be used.
Detection of biomarkers, in particular their quantification and validation, are necessary for understanding the senescence processes (diagnostic biomarkers), monitoring of the rate of senescence (prognostic and predictive biomarkers) and the possible use of appropriate therapy intervention (pharmacodynamic biomarkers).
The identification and selection of reliable biomarker(s), and the use of reproducible methods could help to better understanding of complex web of senescence and aging processes, but it will also open some new questions.
Despite new findings at the cellular and molecular level the understanding the aging process is still limited.
